# Strategies for maintaining and strengthening the health care workers during epidemics: a scoping review

**DOI:** 10.1186/s12960-023-00844-2

**Published:** 2023-08-01

**Authors:** Sadra Valiee, Zahra Zarei Jelyani, Mohammad Kia, Ali Jajarmizadeh, Sajad Delavari, Naseh Shalyari, Milad Ahmadi Marzaleh

**Affiliations:** 1 Student Research Committee, School of Medicine, Shiraz University of Medical Sciences Shiraz, Shiraz, Iran; 2 Department of Health Management and Economics, School of Public Health, Tehran University of Medical Sciences, Tehran, Iran; 3 School of Health Management and Information Sciences, Health Human Resources Research Center, Shiraz University of Medical Sciences, Shiraz, Iran; 4 Department of Health in Disasters and Emergencies, School of Health Management and Information Sciences, Shiraz University of Medical Sciences, Shiraz, Iran

**Keywords:** Maintaining, Strengthening, Human resources, Health care workers (HCWs), Epidemics, Scoping review

## Abstract

**Introduction:**

During epidemics such as COVID-19, healthcare workers (HCWs) face several challenges, leading to a shortage and weakening of human resources. To address this issue, employing effective strategies is essential in maintaining and strengthening human resources during outbreaks. This study aimed to gather and classify strategies that could retain and strengthen human health resources during epidemics.

**Methods:**

In this scoping review, all studies published about strategies for maintaining and strengthening HCWs in epidemics were collected from 4 international databases, including PubMed, Embase, Scopus, and Web of Science. The English language articles published after 2000 up until June 2022 recommended specific strategies regarding the research question. Then, they were analyzed and classified according to thematic analysis based on Braun and Clarke 6 phases protocols.

**Results:**

In total, 9405 records were screened, of which 59 articles were included, and their full texts were reviewed. Fifty factors were identified and classified into five themes: Instruction, Protection, Supporting, Caring, and Communication. Most of the suggestions were conducted in high-income countries and related to the Supporting theme.

**Discussion:**

The majority of strategies discussed in the literature addressed only one or two aspects of human resources. This study provides a holistic perspective on these issues by providing a thematic map of different strategies for strengthening and maintaining HCWs during epidemics. Considering the multidimensionality of human nature, it is suggested that policymakers and managers of health systems provide facilities that simultaneously address a wide range of needs.

**Supplementary Information:**

The online version contains supplementary material available at 10.1186/s12960-023-00844-2.

## Introduction

The World Health Organization (WHO) reported a pneumonia outbreak on December 31st, 2019, that seemed to be associated with a seafood wholesale market in Huanan, Wuhan Province, China [1]. It was identified in early January 2020 that the pathogenic agent was a novel beta-coronavirus with over 85% similarity to a bat SARS coronavirus (SARS-CoV). SARS-CoV-2 and Coronavirus disease 2019 (COVID-19) have been named the new virus and the disease, respectively. The epidemic of COVID-19 virus has spread globally through human-to-human transmission and was declared an international health emergency on January 30th, 2020. [1, 2]. According to official figures, as of September 7th, 2022, 230 countries, areas, or territories were affected, with 603,711,760 confirmed cases and 6,484,136 deaths [3].

In the wake of the epidemics, all capacities have been challenged in preparing for and responding to the disease. Different strategies are used in various countries to combat viral transmission. Nevertheless, the relative success of these strategies depends on the resilience of health systems across all levels. As a result of the COVID-19 pandemic, most health systems have revealed their limitations [4].

According to the WHO's documents, the health workforce is considered one of the health systems' main blocks and key input components [5]. It is a crucial element for the resilience of health systems in facing various natural and human disasters [6]. Also, these events, including epidemics, are typically followed by an increase in healthcare workers' mortality, injury, and disease, along with a deterioration of healthcare systems' human resources [7].

In the aftermath of epidemics, healthcare workers (HCWs) have to deal with the incredible pressure of physical and psychological issues [8]. From January 2020 to May 2021, mixed analytical approaches in WHO's report estimated a range between 80,000 to 180,000 (with a central population-based estimate of 115 500) mortalities among HCWs from COVID-19 [9]. While HCWs represent approximately 1–3% of the population in different countries, about 14% of COVID-19 cases involved HCWs. Furthermore, this ratio can be as high as 35% in some countries [10].

Around the globe, it is estimated that stress, anxiety, depression, insomnia, anger, and fear represent only a tiny minority of all reported injuries among HCWs [11, 12]. Additionally, the medical staff is faced with a double burden of work challenges and psychological stress caused by increased workload, inadequate protective equipment, the observation of colleagues and patients dying, the fear of contracting and transmitting diseases to their family members, the necessity of tolerating quarantine as solitary confinement and social isolation, as well as the dilemma of allocating drugs and hospital equipment to patients in the absence of their family members [12–22].

In these circumstances, many HCWs decide to cease employment. This problem has been noted in the United States [23] and is expected to worsen in low-income countries [24]. Various factors lead to the shortage of HCWs, some of which include their decision to quit their job, life-threatening infections, and the mortality of HCWs. This vicious cycle places renewed strain on the other organization's resources and results in the health systems' collapse [25, 26].

Therefore, one of the most critical issues during epidemic diseases is retaining and strengthening human health resources [27]. While many scattered approaches have been proposed for the maintenance and reinforcement of human resources during epidemics, it is required to conduct a comprehensive study with a functional thematic analysis of these strategies. In this study, we gathered and evaluated potential strategies to improve and sustain human health resources during epidemics using the scoping review methodology.

## Methods

**Fig. 1:**
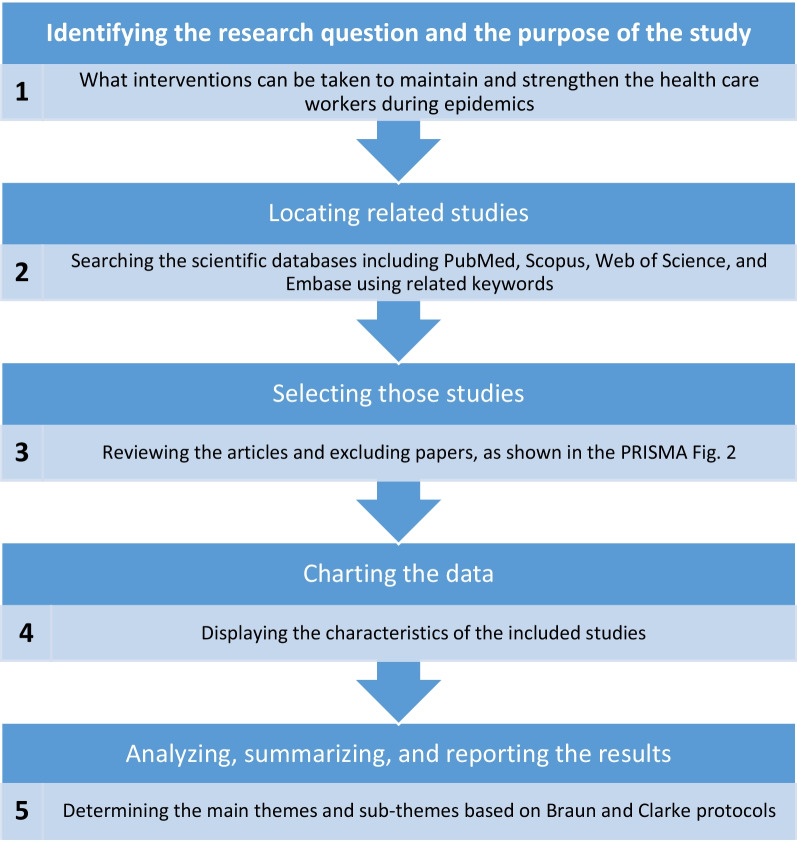
Scoping review methodological steps

This scoping study aims to draw attention to the scope, range, and nature of research activities, describing their results and synthesizing those findings into strategies for maintaining and strengthening HCWs during epidemics [28]. Because there have only been a few comprehensive studies in this field and the research question covers a wide range of topics, a scoping review was chosen as the preferred method for this study. In this regard, the five-step approach proposed by Arksey and O'Malley in 2005 has been utilized. Specifically, these steps involved: (1) identifying the research question and the purpose of the study; (2) locating related studies; (3) selecting those studies; (4) charting the data; and (5) analyzing, summarizing, and reporting the results [28]. More details on these steps can be reviewed in Fig. 1.

### Search strategy

This study's research question was "What strategies can be used to maintain and strengthen the health care workers during epidemics?” The investigation was conducted using international databases, including PubMed, Embase, Scopus, and Web of Science. There are three components to the research question: "maintaining and strengthening", "the health care workers", and "epidemics". Upon completion of the primary search, related studies were reviewed to determine the final keywords for searching. The "AND" operator was employed between each component, and the "OR" operator was also used between synonymous terms. The documents' titles, abstracts, and keywords were searched for relevant results. Also, Mesh Terms were utilized to search for publications in the PubMed database. An illustration of the search strategy and keywords used in searching is presented in Table 1. A total number of 8737 documents were found via searching "strategy" and keywords. In order to manage the references, we employed the ENDNOTE edition X9 program.

**Table 1:** Search strategy on international databases

PIO	#1 AND #2 AND #3	Strategy
P	Endemic **OR** Epidemic **OR** Pandemic **OR** Disease outbreak	#1
I	Retention **OR** Sustain **OR** Maintain **OR** Resil* **OR** Burnout	#2
O	Physician **OR** Nurse **OR** Healthcare Provider **OR H**ealthcare Professional **OR** Medical Staff **OR** Health Personnel **OR** Health Workforce	#3

### Selection process

Inclusion and exclusion criteria, including time, language, and subject area are available in Table 2. The present study covers English papers published over 22 years (2000–2022), totaling 8477 publications. The duplicate publications were removed from this list by eliminating 2875 articles. All titles were checked, and those related to the research question that matched the inclusion criteria were selected. Then, the abstracts were reviewed, and the papers that met the inclusion criteria were chosen. Overall, the full text of each research paper was reviewed and analyzed after evaluating the titles and abstracts of 5602 based on inclusions and exclusions criteria with emphasis on two criteria:

A) The simultaneous presence of three aspects of "maintaining and strengthening", "epidemics", and "health care workers".
B) The proposal of specified and precise strategies.

**Table 2:** Inclusion and exclusion criteria

Criterion	Inclusion	Exclusion
Time period	2000–June 2022	Any study outside these dates
Language	English	Non-English
Subject area	Related to the research question	Non-related to the research question

**Fig. 2:**
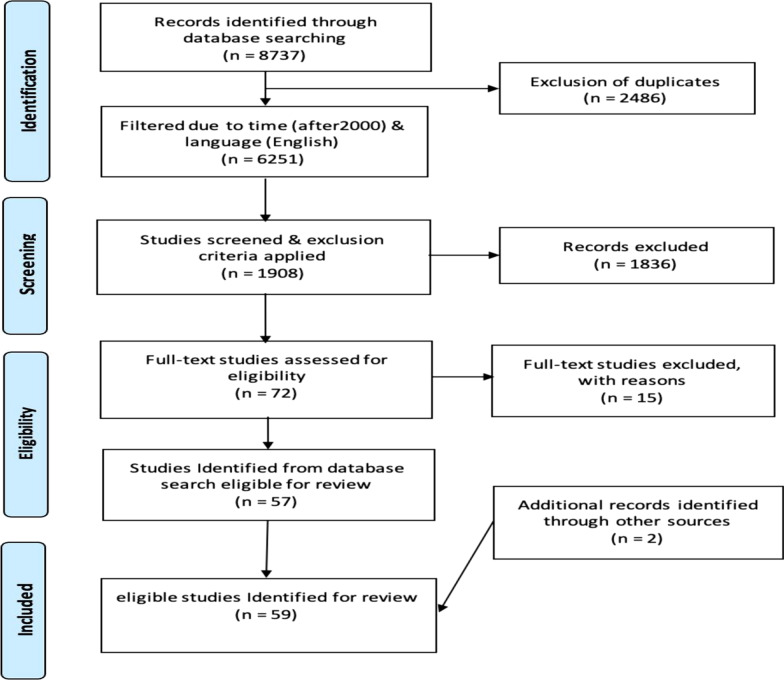
Selection process (PRISMA flow diagram)

As a precaution, to reduce the error possibility, all titles and abstracts were reviewed independently by two researchers (S.V. and A.J.). In case of disagreement, a third researcher (Z.Z.) would make the final decision. Six studies out of 72 were excluded due to the unavailability of their full text. Also, nine studies were excluded for their irrelevancy to the research question. Finally, by incorporating two studies from hand-search studies, the final 59 studies were included. Figure 2 illustrates the summary of this process.

### Data extraction and analysis

The relevant data were gathered from the papers and summarized in a table. This table, which is in Additional file 1: Appendix 1, includes the title of the research, country, type of study, focus, and relevant findings in the research. The results were categorized and integrated by thematic analysis based on Braun and Clarke's 6-phase framework, which is an inductive approach. These phases include familiarizing with data, generating initial codes, searching for themes, reviewing themes, defining themes, and writing up the report [29].

## Results

In this study, we examined the results of articles that provided strategies for maintaining, strengthening, and sustaining human resources during epidemic adversities. More studies were conducted in the United States and the United Kingdom. One study represented dentists as the study population, while 46 concerned general healthcare workers. Correspondingly, five studies focused on physicians, and eight were based on nurses. These details and other characteristics of included studies can be found in Table 3. Furthermore, based on the thematic analysis of the proposed strategies, 15 sub-themes were compiled from five main themes: Instruction, Protection, Supporting, Caring, and Communication (shown in Table 4).

**Table 3:** Characteristics of included literature

Characteristics	Type	Number of studies
Countries’ economic status	High-income	25
	Upper-middle income	2
	Lower-middle income	5
	Low income	0
	Not mentioned country	27
Total		59
Study population	HCWs	45
	Physicians	5
	Nurses	8
	Dentist	1
Total		59

**Table 4:** Themes, sub-themes and strategies

Theme	Subthemes	Strategies	Number of references
Instruction	Creating educational networks	Creating educational networks to share successful experiences and the most up-to-date information about the disease, ways of prevention, protection, and treatment measures	7
	Providing educational programs	Meeting the educational needs of HCWs in various aspects, including lifestyle, mental health, mental exercises such as mindfulness practices or meditation, clinical skills innovation implementation stages, and infection control methods Use of webinars, software, QR codes, text messages or presented websites, and digital packages to facilitate staff educating Provide preparedness plans to deal more with emergencies	24
Protection	Virtual practicing	Use of telemedicine for in-home screening Referral stations to the medical staff via video communication Using Tele-ICU	7
	Protective measures and equipment supply	Supplying personal protective equipment Providing vaccines and diagnostic tests for medical staff and their families Establishing a suitable place to quarantining sick and suspicious HCWs Disinfection of surfaces regularly Considering transportation facilities for HCWs to prevent disease transmission	20
	Revising and adjusting work shifts	Using special staffing models Not using vulnerable people (elderly, physically weak, etc.) in the frontline Reorganizing work shifts to reduce the spread of disease among HCWs	4
	Early detection	Continuous monitoring of the condition of the HCWs for early detection Quarantining the suspicious or sick staff	3
Supporting	Psychological support	Organizing a specialized psychological team to provide counseling Establishing psychological first aid platform to provide counseling Celebrating patients' recovery to boost positive energy Allocating space to keep alive the memory of deceased HCWs Arranging breathing exercises to reduce work-related stress Helping the work community by make meaning of the event, and then helping personnel to look hopefully to the future Sending supportive and motivational messages to patients, managers, and prominent social activists Limit exposure to disaster related and other negative media	21
	Peer support	Creating a culture of support and a sincere atmosphere among HCWs to support each other Holding Schwartz rounds	20
	Welfare facilities	Considering the appropriate space and time for HCWs to rest Providing parking, lodging & allocating centers for child care staff Providing easy access to healthy water and food for HCWs	12
	Professional support	Delegation of powers for decentralized decision-making Involvement of HCWs in the decision-making process Establishing organizational justice and creating a flexible organizational culture to increase teamwork atmosphere Rewards and financial support Design resilience model to anticipate, plan and deter responder risk HCW support during disputes with patients' families Facilitating communication between the patient and his family to reduce the workload caused by the supportive role of the treatment staff	11
	Workload support	Recruitment of new employee Developing a clear and realistic plan to reduce workload Deprioritize non-essential projects to reduce workload Organizing volunteer forces to compensate for labor shortages Strategic distribution of human resources by reviewing the work-shift program Using a support team consisting of specialists ready to work in various fields, including palliative medicine and dental team	15
Caring	Psychological care services	Using health applications to provide medical services Holding group therapy sessions and using special treatment techniques such as CBT Retraining counselors and psychologists and teach them treatment techniques to provide adequate human resources Use Medications for depression, anxiety, sleep disorders, or a combination of any or all of these	10
	Non-psychological care services	Prioritizing HCWs and their families in treatment and drug allocation Providing services through narrative medicine	3
Communication	Active communication	Establishing explicit, effective, and honest communication from the manager with the staff Managers' personal visits to the departments and have regular sessions to check and compensate for the shortcomings various areas	15
	Passive communication	Creating a communication network to receive feedback Setting up hotlines to hear the HCWs' concerns	5

### Instruction

#### Creating educational networks

An educational network can be set up at the local, national, and international levels to facilitate the sharing of successful experiences and updated information regarding the spread of disease, protection, prevention, and therapeutic measures [18, 30–35]. For instance, such content can be created on social media and under the supervision of health education organizations.

#### Providing educational programs

The educational requirements of the medical community can also be addressed in diverse areas, including lifestyle skills [21, 35–39], as well as mental health issues [8, 13, 14, 17, 21, 31, 33, 35–49], mental skills training such as mindfulness practices and meditation [21, 37, 39, 45], as well as clinical skills [14, 23, 39], and infection control methods [8, 12, 21, 30, 35]. Programs like these can be offered through online webinars [38, 50], smartphone applications [21, 49], QR codes [33], or even digital packages [51]. In addition, HCWs, particularly their families, can learn to adapt to emergency preparedness programs from reputable websites [35, 52].

### Protection

#### Virtual practicing

The risk of infection among physicians can be reduced with remote care models such as telemedicine. These establish a social distance between HCWs and patients and prevent hospital overcrowding. This technology allows patients to receive initial screening and treatment without being physically present [14–16, 21, 53–55].

#### Protective measures and equipment supply

The availability of Personal Protective Equipment (PPE), including N95 masks or gloves, is one of the critical factors that contribute to health care workers' willingness to serve in emergencies and epidemics [8, 17, 23, 30, 35, 36, 41, 49, 52, 56–61]. Further, it is essential to provide vaccines and diagnostic tests to healthcare providers and their families [23, 30]. It may also be necessary to reprocess protective equipment [59] and establish a healthcare facility for isolating sick or suspicious individuals [30, 44]. Some effective measures can be taken to prevent infection of HCWs, including the use of infection control checklists [24], disinfecting surfaces regularly (based on the microorganism and the latest information) [56], and providing transportation facilities for HCWs during outbreaks [23, 44, 58].

#### Revising and adjusting work shifts

One method to reduce the shortages of healthcare workers during epidemics is using staffing models informed by epidemiology [8]. Additionally, to minimize injuries among medical staff, human resources might be allocated strategically, and it is suggested not to place vulnerable individuals (the elderly or those with underlying diseases) on the front lines [8, 30, 47, 56].

#### Early detection

In order to ensure the safety of HCWs, it is prudent to monitor their health status and implement measures to quarantine patients who appear to be positive or suspicious [8, 27, 62].

A comprehensive model was done in a hospital in Singapore based on three phases: "(1) enforcing reporting of HCWs with acute respiratory illness (ARI) to the institution’s staff clinic for monitoring; (2) conducting ongoing syndromic surveillance to obtain early warning of potential clusters of COVID-19; and (3) outbreak investigation and management " which is a successful example of this strategy [27].

### Supporting

#### Psychological support

It is recommended to set up a special team of psychiatrists and psychologists to reduce fatigue and burnout among front-line employees [36, 41, 46, 61, 63]. Several studies have proposed using psychological first aid platforms [17, 39] to address emergency mental issues of HCWs [21, 31, 34, 40, 44, 50, 64]. Additionally, organizations can provide psychological support by celebrating patients' recovery, allocating space to honor the memory of deceased healthcare workers, and arranging breathing exercises to reduce work-related stress [12]. Making meaning of the event and helping personnel look hopefully to the future can boost the work community [21, 35, 37, 61]. Instilling a sense of empathy with the front-line workers and sending supportive and motivational messages from patients, managers, and prominent social activists are suggested, too [21, 32, 33, 64]. Also, taking measures towards limiting exposure to disaster and other related negative media may prevent more psychological injuries for HCWs [35].

#### Peer support

To alleviate the psychological burden caused by crises, cultivating a communicational network and a cordial atmosphere among healthcare workers is suggested. This network enables them to support one another, express empathy, and involve themselves more frequently. In addition, this network offers peer support during quarantine. [12, 19–21, 30–34, 36, 39, 41, 42, 45, 46, 48, 61, 63, 65, 66].

#### Welfare facilities

As healthcare workers perform vital duties in epidemic situations, ensuring their physical and mental well-being is of utmost importance. Several recommendations have been made in order to accomplish this purpose. These include sufficient holidays, break times and breakrooms, and easy access to drinking water and food during the working shifts. Locating centers for the care of children of HCWs during school closures, besides providing parking spaces and accommodation, are other suggestions of this sub-theme [17, 20, 33–35, 37, 39, 42, 44, 45, 58, 66].

#### Professional support

It is suggested that HCWs be involved in decision-making and empowered to delegate the requisite authority for decentralized decision-making [14, 37, 43]. Several approaches have been proposed in the literature to strengthen and maintain human resources, such as designing a resilience model to anticipate, plan and deter responder risk [21, 61], creating organizational justice besides promoting a flexible organizational culture based on a teamwork atmosphere [21, 37, 38, 43, 61], offering rewards and financial support [48, 67], and extending support to HCWs during times of conflict with patients' families [23]. Furthermore, it is also conceivable for a third party to facilitate or mediate communication between the patients and their families, which will relieve HCWs and especially nurses of work-related stress [48].

#### Workload support

A strategic allocation of human resources contributes enormously to compensating for labor shortages and reducing workloads and burnout. This can be ensured by developing a clear and realistic plan [19, 31, 32, 43], deprioritizing non-essential projects [60], requiring new employees from a wide variety of front-line clinicians [60, 61], organizing the volunteers [17, 30], modifying work-shift schedules, and taking measures to provide remote care [17, 38, 39, 42, 47]. In an emergency, it is reasonable to assemble a support team comprising ready-to-work specialists with experience in health-related fields, such as pharmacists, dentists [8, 23, 34, 68].

### Caring

#### Psychological care services

Psychological trauma requires the commitment of supervisors and managers to provide treatment. In this regard, it is advisable to institute protocols for ongoing assessment and evaluation of healthcare workers' mental health, besides considering the deployment of dedicated teams for early intervention and treatment of traumatized individuals [14, 33, 36, 39, 41, 48, 66]. Also, some other strategies are proposed, such as providing medical applications to screening and delivering specialized medical services [39, 46, 69], conducting group therapy sessions or medication-based therapy [33, 39], and employing special treatment techniques such as cognitive behavioral therapy (CBT) to solve and deal with psychological ailments [70]. In order to provide the human resources for these approaches, counselors and psychologists need to be well-versed in treatment practices [70].

#### Non-psychological care services

The two strategies outlined in this sub-theme aim to provide particular treatment and drug allocation to HCWs and their families and use narrative medicine to provide services for them [32, 37, 59].

### Communication

#### Active communication

It has been ascertained that explicit, effective, and honest communication among managers and HCWs results in increased security, unity, teamwork [37, 47, 61], and resilience in the HCWs [14, 21, 31, 42]. Because HCWs expect to be understood and acknowledged, managers are advised to be aware of their concerns and respond appropriately. Also, enhancing communication with HCWs is crucial in ensuring a resilient environment [18, 35, 67]. Specifically, hospital managers have been asked to visit hospital wards [33, 48]. Moreover, regular sessions [21] and active communication are considered for problem resolution [21, 23, 27].

#### Passive communication

Initiating hotlines to respond to HCWs' needs and queries swiftly and effectively [11, 18, 56] and developing a communication network to obtain feedback are other suggested strategies for this sub-theme [58, 71].

## Discussion

The purpose of this study was to collect data on strengthening and retaining methods of HCWs in epidemics. Strategies proposed in most papers have not been studied in specific geographical areas, and the target population of most cases was all HCWs**.** In sum, the findings show that the three categories of "Instruction", "Protection", and "Supporting" can prevent harm. Conversely, strategies mentioned in the "Caring" and "Communication" categories can address health care workers' possible injuries. Furthermore, the most suggested strategies are related to the themes of "Instruction", "Protection", and "Supporting", respectively, and the ones mentioned in "Treatment" and "Feedback" are significantly less than the previous three themes.

Considering this difference, prevention must be prioritized over treatment in strengthening and maintaining health workers. In addition, among the sub-themes, the most frequently discussed factor were Training Programs, Protection and Equipment Provision, Professional Support, Peer Support, and the Intelligent Use of Human Resources, respectively. However, due to the difference in the context of different regions, it is preferable that managers and policymakers choose and implement the suggested strategies based on their specific circumstances.

Healthcare systems face new challenges as a new disease emerges and spreads rapidly across different nations. In situations like that, it is essential to prepare the system and its employees for unfamiliar circumstances by educating the staff with the help of various formal and informal instructions. When addressing an epidemic crisis, physicians and nurses must acquire the latest information about that disease. Aside from this, due to the high workload and psychological stress that exists during this time, staff can be trained in various fields, especially in developing positive lifestyles and personal resilience [36]. Moreover, it may be feasible to provide tailored educational packages for HCWs through digital packages [51]. Furthermore, with the emergence of social networks in communities, it is recommended to use these platforms to share HCWs' experiences and knowledge. [18, 34].

As HCWs are on the frontline of managing epidemics, they may quickly become infected. Therefore, one of the most practical and vital strategies is safeguarding and reducing the risk of disease transmission among them. Under these circumstances, personal protective equipment (PPE) is essential for healthcare workers [56]. The early detection of infected HCWs is another critical issue in this context. Accordingly, Singapore's most significant medical hospital has implemented a three-stage public reporting model. This model includes establishing a self-reporting system based on suspicious cases, identifying and screening persons in contact with suspected cases, and initiating quarantine alongside necessary treatment for sick employees. In this instance, only 14 of 4411 hospital staff members tested positive for COVID-19 over 16 weeks, and these results indicate that the process has been so effective. Additionally, Virtual Practicing is another suggestion that can cut down face-to-face visits and provide high-quality care to patients, reducing the spread of diseases among HCWs [55]. The advantage of this strategy is its overlapping with the themes of support and treatment by reducing workload and facilitating the provision of medical services to health personnel in need [27]. There are, however, several limitations to this intervention. These include the high costs, the requirement of building multiple infrastructures, the potential for system disruptions, and the current workforce's reluctance to undergo significant changes [54].

Human resources need to cope with the new outbreak and be prepared to take protective measures to reduce the risk of infection. However, their organizations, managers, and peers will always require support and assistance. Consequently, the majority of the strategies are related to supporting healthcare workers. HCWs need different forms of support during epidemics, including psychological support, welfare facilities, and professional assistance, for which managers are primarily responsible. It is unavoidable that managers create teams of psychological experts and provide counseling services to HCWs to deal with the effects of the existing workload [34]. It is also notable that HCWs can support and help each other independently of their managers to reduce the stress of these sensitive situations [42], and it is without a doubt the role of management to foster such a supportive culture and sincere ambiance [49]. Another key strategy is the wise use of human resources and their strategic distribution by reviewing shift schedules and enlisting volunteer workers in the HCWs' support [30, 47]. Considering that many strategies have been proposed and implemented regarding the psychological support of HCWs, a systematic review is recommended in this context.

Considering the above points, the proposed elements for the three themes of Instruction, Protection, and Supporting are more closely aligned with preventing injury. The reality is that some HCWs are adversely affected by severe injuries that require appropriate treatment when they become physically or mentally ill. Meanwhile, mental health issues, which are more common and more challenging to diagnose, are suggested to be addressed in greater detail. As an additional measure, improving medical facilities for HCWs' families' needs can be quite effective [57].

If we take a comprehensive approach regarding the maintenance and strengthening of healthcare workers, constant communication is one of the unique features of this issue. Communication is essential for gaining insight into the present state, evaluating the implemented strategies, and applying alternative plans as indicated. Moreover, a point worth emphasizing is that communication should not be a cross-cutting procedure but a permanent process. As a result, due to the importance of the issue, it is recommended to facilitate this process using the proposed strategies to receive active and passive Communication. In essence, this is a complementary component of the strategies in the other themes and a way to evaluate them. To communicate, it appears feasible to establish a hotline [18, 56] to listen to HCWs' needs and provide assistance, as it does not require a vast infrastructure or high costs. It is essential to consider many factors in this strategy, including establishing a transparent communication program, providing necessary human resources, and following the confidentiality principle [11]. In addition, it is mentioned in recent human resource management literature that hierarchical leadership that is existed in traditional organizations has adverse effects on HCWs, and it can lead to putting individuals to blame rather than inspiring collectively responsible morale [72]. Therefore, it is noticeable to consider active and passive communication to move on from traditional organizations with hierarchical leadership to organizations with flat structures.

Generally, conducted studies examined a variety of strategies in a scattershot manner. Our study categorized these strategies to summarize them into thematic maps and categories. It is indisputable that humans are multidimensional beings with diverse needs. However, it is worth noting that most scientific studies have focused on only one or two aspects of their needs. In order to meet these various needs, our study has looked at this issue more comprehensively, and the findings may assist managers and policymakers in designing more effective programs to strengthen and maintain healthcare workers during epidemics.

One of the most intriguing points about this collection of studies is that some of the findings contradict one another. For example, in the study of Elsafty et al. in Egypt [67], financial incentives proved to be an effective method to strengthen and sustain human resource capacity during epidemics. Despite this, in another study conducted by Martin et al. in the United States, increased pay was not an effective motivator toward the willingness of HCWs to work [52].

Consequently, it is recommended that further research be done to evaluate the feasibility of these strategies and to determine their effectiveness. Also, due to the limited resources and the need to save time and costs, it is essential to conduct cost-effectiveness studies. Furthermore, since most studies have been conducted in countries with high incomes, we recommend that strategies available in lower-income countries be evaluated for their effectiveness and feasibility. In addition, systematic reviews are suggested to analyze conflicting findings and investigations that cover multiple themes of instruction, protection, supporting, and caring.

## Limitations

Numerous strategies have been suggested for strengthening and maintaining human resources. But, most of them have not been implemented yet, or if they did, the required infrastructures and implementation process have not been reported in detail. Failure to examine the long-term effects of interventions, lack of a control group, and lack of cost-effectiveness studies of the suggested strategies were other weaknesses of the available studies.

## Conclusion

We have outlined and summarized strategies to promote the strength and maintenance of human resources during epidemics. Based on the analysis, the strategies were categorized into five main themes: Instruction, Protection, Supporting, Caring, and Communication. Apart from them, 15 sub-themes were classified, including the Creation of Educational Networks, Provision of Educational Programs, Virtual Practicing, Protective Measures and Equipment Supply, Revisions and Adjustment of Work Shifts, Early Detection, Psychological Support, Peer Support, Welfare facilities, Professional Support, Workload Support, Psychological Care Service, Non-psychological Care Service, as well as the Provision of Active and Passive Communication. Developing our human resources in the face of epidemics requires a holistic approach, which means that focusing separately on each of the following themes is not helpful. Furthermore, it is proposed that managers and decision-makers devise strategies that align with their context and cover broader themes.

## Supplementary Information

**Additional file 1. Appendix 1.** Search strategy on international databases. **Appendix 2.** Data summary and relevant findings in reviewed literature.

## Data Availability

Data of this research are available and could be sent upon contact with the corresponding author.

## References

[1] Worobey M, Levy JI, Malpica Serrano L, Crits-Christoph A, Pekar JE, Goldstein SA (2022). The Huanan seafood wholesale market in Wuhan was the early epicenter of the COVID-19 pandemic. Science.

[2] WHO. Press conferences on COVID-19 and other global health issues.

[3] WHO. WHO Coronavirus (COVID-19) Dashboard 2022. https://covid19.who.int/.

[4] Haldane V, De Foo C, Abdalla SM, Jung A-S, Tan M, Wu S (2021). Health systems resilience in managing the COVID-19 pandemic: lessons from 28 countries. Nat Med.

[5] World Health Organization. Everybody's business--strengthening health systems to improve health outcomes: WHO's framework for action. 2007.

[6] Fridell M, Edwin S, Von Schreeb J, Saulnier DD (2020). Health system resilience: what are we talking about? A scoping review mapping characteristics and keywords. Int J Health Policy Manag.

[7] World Health Organization. Health workforce 2030: towards a global strategy on human resources for health. 2015.

[8] Mascha EJ, Schober P, Schefold JC, Stueber F, Luedi MM (2020). Staffing with disease-based epidemiologic indices may reduce shortage of intensive care unit staff during the COVID-19 pandemic. Anesth Analg.

[9] World Health O. The impact of COVID-19 on health and care workers: a closer look at deaths. Geneva: World Health Organization; 2021 2021. Contract No.: WHO/HWF/WorkingPaper/2021.1.

[10] World Health Organization. Keep health workers safe to keep patients safe: WHO. News release. 2020;17.

[11] Geoffroy PA, Le Goanvic V, Sabbagh O, Richoux C, Weinstein A, Dufayet G (2020). Psychological support system for hospital workers during the covid-19 outbreak: rapid design and implementation of the Covid-Psy Hotline. Front Psychiatry..

[12] Gujral H, Rushton CH, Rosa WE (2020). Action steps toward a culture of moral resilience in the face of COVID-19. J Psychosoc Nurs Ment Health Serv.

[13] Maunder RG, Leszcz M, Savage D, Adam MA, Peladeau N, Romano D (2008). Applying the lessons of SARS to pandemic influenza: an evidence-based approach to mitigating the stress experienced by healthcare workers. Can J Public Health.

[14] Restauri N, Sheridan AD (2020). Burnout and posttraumatic stress disorder in the Coronavirus Disease 2019 (COVID-19) pandemic: intersection, impact, and interventions. J Am Coll Radiol.

[15] Moazzami B, Razavi-Khorasani N, Dooghaie Moghadam A, Farokhi E, Rezaei N (2020). COVID-19 and telemedicine: immediate action required for maintaining healthcare providers well-being. J Clin Virol.

[16] Labrague LJ, de Los Santos J (2020). COVID-19 anxiety among frontline nurses: predictive role of organisational support, personal resilience and social support. J Nurs Manag.

[17] Santarone K, McKenney M, Elkbuli A (2020). Preserving mental health and resilience in frontline healthcare workers during COVID-19. Am J Emerg Med.

[18] Rangachari P, Woods JL (2020). Preserving organizational resilience, patient safety, and staff retention during COVID-19 requires a holistic consideration of the psychological safety of healthcare workers. Int J Environ Res Public Health.

[19] Shechter A, Diaz F, Moise N, Anstey DE, Ye S, Agarwal S (2020). Psychological distress, coping behaviors, and preferences for support among New York healthcare workers during the COVID-19 pandemic. Gen Hosp Psychiatry.

[20] Saqib A, Rampal T (2020). Quality improvement report: setting up a staff well-being hub through continuous engagement. BMJ Open Qual..

[21] Heath C, Sommerfield A, von Ungern-Sternberg BS (2020). Resilience strategies to manage psychological distress among healthcare workers during the COVID-19 pandemic: a narrative review. Anaesthesia.

[22] Pourahmadi M, Delavari S, Delavari S (2020). The role of empathy in full-scale battle of medical and paramedical learners against COVID-19. Iran J Med Sci.

[23] Taylor BL, Montgomery HE, Rhodes A, Sprung CL (2010). Protection of patients and staff during a pandemic. Intensive Care Med.

[24] Soma M, Jacobson I, Brewer J, Blondin A, Davidson G, Singham S (2020). Operative team checklist for aerosol generating procedures to minimise exposure of healthcare workers to SARS-CoV-2. Int J Pediatr Otorhinolaryngol.

[25] Pavignani E (2011). Human resources for health through conflict and recovery: lessons from African countries. Disasters.

[26] Vardarlıer P (2016). Strategic approach to human resources management during crisis. Procedia-Soc Behav Sci..

[27] Wee LE, Sim XYJ, Conceicao EP, Aung MK, Goh JQ, Yeo DWT (2020). Containment of COVID-19 cases among healthcare workers: the role of surveillance, early detection, and outbreak management. Infect Control Hosp Epidemiol.

[28] Arksey H, O'Malley L (2005). Scoping studies: towards a methodological framework. Int J Soc Res Methodol.

[29] Braun V, Clarke V (2006). Using thematic analysis in psychology. Qual Res Psychol.

[30] Chersich MF, Gray G, Fairlie L, Eichbaum Q, Mayhew S, Allwood B (2020). Covid-19 in Africa: care and protection for frontline healthcare workers. Glob Health.

[31] Aghili SM, Arbabi M (2020). The COVID-19 Pandemic and the Health Care Providers; What Does It Mean Psychologically?. Adv J Emerg Med..

[32] Wu AW, Connors C, Everly GS (2020). COVID-19: Peer support and crisis communication strategies to promote institutional resilience. Ann Intern Med.

[33] Presti G, Dal Lago B, Fattori A, Mioli G, Moderato P, Sciaretta L (2020). Mental health support to staff in a major hospital in Milan (Italy) during the COVID-19 pandemic: a framework of actions. General Psychiatry.

[34] Akgün KM, Collett D, Feder SL, Shamas T, Schulman-Green D (2020). Sustaining frontline ICU healthcare workers during the COVID-19 pandemic and beyond. Heart Lung.

[35] Morganstein JC, Flynn BW (2021). Enhancing psychological sustainment & promoting resilience in healthcare workers during COVID-19 & beyond: adapting crisis interventions from high-risk occupations. J Occup Environ Med.

[36] Balasubramanian A, Paleri V, Bennett R, Paleri V (2020). Impact of COVID-19 on the mental health of surgeons and coping strategies. Head Neck.

[37] Giordano F, Cipolla A, Ungar M (2022). Building resilience for healthcare professionals working in an Italian red zone during the COVID-19 outbreak: a pilot study. Stress Health.

[38] Avendano F, Hoyos A, Cruz J, Vizcarra F, Lidice K, Grandon R. Burnout syndrome prevention in nursing at pandemic covid-19: a systematic review. Revista De Salud Publica-Cordoba. 2021:48–59.

[39] Pollock A, Campbell P, Cheyne J, Cowie J, Davis B, McCallum J (2020). Interventions to support the resilience and mental health of frontline health and social care professionals during and after a disease outbreak, epidemic or pandemic: a mixed methods systematic review. Cochrane Database of Syst Rev..

[40] Schreiber M, Cates DS, Formanski S, King M (2019). Maximizing the resilience of healthcare workers in multi-hazard events: lessons from the 2014–2015 Ebola response in Africa. Mil Med.

[41] Albott CS, Wozniak JR, McGlinch BP, Wall MH, Gold BS, Vinogradov S (2020). Battle buddies: rapid deployment of a psychological resilience intervention for health care workers during the COVID-19 pandemic. Anesth Analg.

[42] Lefèvre H, Stheneur C, Cardin C, Fourcade L, Fourmaux C, Tordjman E (2020). The Bulle: Support and prevention of psychological decompensation of healthcare workers during the trauma of the COVID-19 epidemic. J Pain Symptom Manag.

[43] Maunder RG, Lancee WJ, Mae R, Vincent L, Peladeau N, Beduz MA (2010). Computer-assisted resilience training to prepare healthcare workers for pandemic influenza: a randomized trial of the optimal dose of training. BMC Health Serv Res.

[44] Wei E, Segall J, Villanueva Y, Dang LB, Gasca VI, Gonzalez MP (2020). Coping with trauma, celebrating life: reinventing patient and staff support during the COVID-19 pandemic. Health affairs (Project Hope).

[45] Roney LN, Beauvais AM, Bartos S (2020). Igniting change: supporting the well-being of academicians who practice and teach critical care. Crit Care Nurs Clin North Am.

[46] DePierro J, Katz CL, Marin D, Feder A, Bevilacqua L, Sharma V (2020). Mount Sinai's center for stress, resilience and personal growth as a model for responding to the impact of COVID-19 on health care workers. Psychiatry Res.

[47] Billings J, Greene T, Kember T, Grey N, El-Leithy S, Lee D (2020). Supporting hospital staff during COVID-19: early interventions. Occup Med.

[48] Owens IT (2020). Supporting nurses' mental health during the pandemic. Nursing.

[49] Otu A, Ebenso B, Okuzu O, Osifo-Dawodu E (2016). Using a mHealth tutorial application to change knowledge and attitude of frontline health workers to Ebola virus disease in Nigeria: a before-and-after study. Hum Resour Health.

[50] Weingarten K, Galván-Durán AR, D’Urso S, Garcia D (2020). The witness to witness program: helping the helpers in the context of the COVID-19 pandemic. Fam Process.

[51] Blake H, Bermingham F, Johnson G, Tabner A (2020). Mitigating the psychological impact of COVID-19 on healthcare workers: a digital learning package. Int J Environ Res Public Health.

[52] Martin SD, Brown LM, Reid WM (2013). Predictors of nurses' intentions to work during the 2009 influenza A (H1N1) pandemic. Am J Nurs.

[53] Doshi A, Platt Y, Dressen JR, Mathews BK, Siy JC (2020). Keep calm and log on: Telemedicine for COVID-19 pandemic response. J Hosp Med.

[54] Rathod N, Rajput A, Fouzia M, Jyothi DB, Patil K (2020). E-ICU's/tele ICU's, it's role, advantages over manual icu's and shortcomings in the current perspective of covid-19 pandemic: a critical review. Int J Cur Res Rev.

[55] Vargheese R, editor Leveraging cloud based virtual care as a tool kit for mitigating risk of exposure during a pandemic. 5th International Conference on Emerging Ubiquitous Systems and Pervasive Networks, EUSPN 2014 and the 4th International Conference on Current and Future Trends of Information and Communication Technologies in Healthcare, ICTH 2014; 2014: Elsevier B.V.

[56] Majeed A, Molokhia M, Pankhania B, Asanati K (2020). Protecting the health of doctors during the COVID-19 pandemic. Br J Gen Pract.

[57] Park YS, Behrouz-Ghayebi L, Sury JJ (2015). Do shared barriers when reporting to work during an influenza pandemic influence hospital workers' willingness to work? A multilevel framework. Disaster Med Public Health Prep.

[58] Ripp J, Peccoralo L, Charney D (2020). Attending to the emotional well-being of the health care workforce in a New York City health system during the COVID-19 pandemic. Acad Med.

[59] Rowan NJ, Laffey JG (2020). Challenges and solutions for addressing critical shortage of supply chain for personal and protective equipment (PPE) arising from Coronavirus disease (COVID19) pandemic – Case study from the Republic of Ireland. Sci Total Environ.

[60] Berkow S, Virkstis K, Herleth A, Whitemarsh K, Rewers L (2020). An executive strategy to support long-term clinician engagement amid the COVID-19 pandemic. J Nurs Adm.

[61] Ralph J, Freeman LA, Ménard AD, Soucie K (2021). Practical strategies and the need for psychological support: recommendations from nurses working in hospitals during the COVID-19 pandemic. J Health Org Manag..

[62] Rivett L, Sridhar S, Sparkes D, Routledge M, Jones NK, Forrest S (2020). Screening of healthcare workers for SARS-CoV-2 highlights the role of asymptomatic carriage in COVID-19 transmission. Elife.

[63] Chesak SS, Perlman AI, Gill PR, Bhagra A (2020). Strategies for Resiliency of Medical Staff During COVID-19. Mayo Clin Proc.

[64] Cheng W, Zhang F, Liu Z, Zhang H, Lyu Y, Xu H (2020). A psychological health support scheme for medical teams in COVID-19 outbreak and its effectiveness. General Psychiatry.

[65] Dewey C, Hingle S, Goelz E, Linzer M (2020). Supporting clinicians during the COVID-19 pandemic. Ann Intern Med.

[66] Donnelly PD, Davidson M, Dunlop N, McGale M, Milligan E, Worrall M (2020). Well-Being during coronavirus disease 2019: a PICU practical perspective. Pediatr Crit Care Med.

[67] Elsafty AS, Ragheb M (2020). The role of human resource management towards employees retention during Covid-19 pandemic in medical supplies sector-Egypt. Bus Manag Stud..

[68] Sacoor S, Chana S, Fortune F (2020). The dental team as part of the medical workforce during national and global crises. Br Dent J.

[69] Alexopoulos AR, Hudson JG, Otenigbagbe O (2020). The use of digital applications and COVID-19. Community Ment Health J.

[70] Rothenberger DA (2017). Physician burnout and well-being: a systematic review and framework for action. Dis Colon Rectum.

[71] Elo S, Kyngäs H (2008). The qualitative content analysis process. J Adv Nurs.

[72] Fernandopulle N (2021). To what extent does hierarchical leadership affect health care outcomes?. Med J Islam Repub Iran.

