# Supplementary material for: Strategies for maintaining and strengthening the health care workers during epidemics: a scoping review
Source: Hum Resour Health. 2023 Aug 1;21:60. doi: 10.1186/s12960-023-00844-2 (PMC10394761; doi:10.1186/s12960-023-00844-2)
Supplement: Supplementary file 1 — Additional file 1. Appendix 1. Search strategy on international databases. Appendix 2. Data summary and relevant findings in reviewed literature. [file 12960_2023_844_MOESM1_ESM.docx]

**Appendiex 1. Search Strategy on International Databases**

| **Date** | **Result** | **Search strategy** | **Database** | **No.** |
| --- | --- | --- | --- | --- |
| 2/06/2022 | 1965 | ("retention"[Title/Abstract] OR "sustain"[Title/Abstract] OR "maintain"[Title/Abstract] OR "burnout, professional"[MeSH Terms] OR "burnout"[Title/Abstract] OR "resil*"[Title/Abstract]) AND ("Health Personnel"[MeSH Terms] OR "Health Workforce"[MeSH Terms] OR "healthcare professional"[Title/Abstract] OR "healthcare provider"[Title/Abstract] OR "medical staff"[Title/Abstract] OR "nurse"[Title/Abstract] OR "physician"[Title/Abstract]) AND ("Endemic Diseases"[MeSH Terms] OR "Disease Outbreaks"[MeSH Terms] OR "endemic"[Title/Abstract] OR "epidemic"[Title/Abstract] OR "pandemic"[Title/Abstract]) | PubMed | 1 |
| 2/06/2022 | 3245 | ( ( TITLE-ABS-KEY ( retention ) OR TITLE-ABS-KEY ( sustain ) OR TITLE-ABS-KEY ( maintain ) OR TITLE-ABS-KEY ( resil* ) OR TITLE-ABS-KEY ( burnout ) ) ) AND ( ( TITLE-ABS-KEY ( physician ) OR TITLE-ABS-KEY ( nurse ) OR TITLE-ABS-KEY ( healthcare AND provider ) OR TITLE-ABS-KEY ( healthcare AND professional ) OR TITLE-ABS-KEY ( medical AND staff ) OR KEY ( health AND personnel jjk) OR KEY ( health AND workforce ) ) ) AND ( ( TITLE-ABS-KEY ( endemic ) OR TITLE-ABS-KEY ( epidemic ) OR TITLE-ABS-KEY ( pandemic ) OR KEY ( disease AND outbreak ) ) ) | Scopus | 2 |
| 2/06/2022 | 2636 | ((TI=(retention OR sustain OR maintain OR resil* OR Burnout ) OR AB=(retention OR sustain OR maintain OR resil* OR Burnout)) AND (TI=(Healthcare professional OR Healthcare provider OR medical staff OR nurse OR physician) OR AB=(Healthcare professional OR Healthcare provider OR medical staff OR nurse OR physician) OR AK=(Health workforce OR Health personnel)) AND (TI=(endemic OR epidemic OR pandemic) OR AB=(endemic OR epidemic OR pandemic) OR AK=(Disease outbreak))) | Web Of Science | 3 |
| 2/06/2022 | 891 | ('health care personnel':kw OR 'health provider':ti,ab,kw OR 'medical staff':ti,ab,kw OR 'health practitioner':ti,ab,kw OR 'health workforce':kw OR physician:ti,ab,kw OR nurse:ti,ab,kw) AND ('endemic disease':kw OR endemic:ti,ab OR epidemic:ti,ab,kw OR pandemic:ti,ab,kw) AND (burnout:ti,ab OR 'professional burnout':kw OR 'resil*':ti,ab,kw OR retention:ti,ab,kw OR sustain:ti,ab,kw OR maintain:ti,ab,kw) | Embase | 4 |

**Appendix2: Data Summary and Relevant Findings in Reviewed Literature**

| **No.** | **Title** | **Country** | **Methods** | **Study Population** | **Focus/aim** | **Relevant Findings** |
| --- | --- | --- | --- | --- | --- | --- |
| 1 | Battle Buddies: Rapid Deployment of a Psychological Resilience Intervention  for Health Care Workers During the Coronavirus Disease 2019 Pandemic | Not mentioned | Review | HCWs | Surveying the psychological effects of coping with Covid-19 on health workers and strategies to increase their resilience | - Provide personal protective equipment - Deploy psychologists - Compile educational guides in various dimensions such as exercise, nutrition, virus transmission methods, etc. - Promote peer support |
| 2 | Mitigating the Psychological Impact of COVID-19 on Healthcare Workers: A Digital Learning Package | United Kingdom | Original | HCWs | Evaluating the results of providing digital learning packages to reduce the destructive psychological effects of Covid-19 on health care workers | - Use digital training packages to provide a variety of training to HCWs |
| 3 | Impact of COVID-19 on the mental health of surgeons and coping strategies | Not mentioned | Review | Physicians | Surveying the factors affecting the mental health of surgeons and providing mechanisms to maintain their mental health at the individual and organizational levels | - Encourage HCWs to practice positive lifestyle behaviors - Use social networks in order to fill emotional gaps - Use Relaxation and Mindfulness techniques - Provide psychological services and follow-up plans with software - Organize specialized teams to provide psychological support to HCWs - Provide personal protective equipment |
| 4 | The Role of Human Resource Management Towards Employees Retention | Egypt | Original | HCWs | Explaining the role of human resource management during the confrontation with Covid-19 in order to prevent its adverse effects on HCWs | - Provide financial benefits - Use supportive leadership method by managers - Facilitate effective communication between management and HCWs |
| 5 | Preserving Organizational Resilience, Patient Safety, and Staff Retention  during COVID-19 Requires a Holistic Consideration of the Psychological Safety of Healthcare Workers | USA | Letter to editor/  Opinion/ Discussion | HCWs | Providing recommendations to maintain the organization's ability to withstand patient safety and staff retention in the high pressure of the Covid-19 crisis | - Use supportive leadership by managers - Create an environment of trust and psychological safety - Expand communication structures between HCWs in order to share their experiences |
| 6 | Protecting the health of doctors during the COVID-19 pandemic | Not mentioned | Letter to editor/  Opinion/ Discussion | Physicians | Surveying the methods of protecting physicians against Covid-19 | - Use telemedicine - Provide personal protective equipment - Train how to use personal protective equipment - Use vulnerable forces away from contaminated environments - Disinfect surfaces regularly |
| 7 | Sustaining frontline ICU healthcare workers during the COVID-19 pandemic and beyond | USA | Letter to editor/  Opinion/ Discussion | HCWs | Providing solutions to retain ICU front-line staff | - Deploy staff strategically in different parts of the hospital - Provide a platform for sharing clinical, managerial, etc. experiences between hospitals - Establish an expert team in the field of psychological issues to serve HCWs - Provide welfare facilities for HCWs |
| 8 | The Use of Digital Applications and COVID‑19 | Not mentioned | Letter to editor/  Opinion/ Discussion | HCWs | Surveying the role of health applications to help health workers deal with mental injuries | - Provide psychological services with mobile health applications |
| 9 | Mount Sinai's Center for Stress, Resilience and Personal  Growth as a model for responding to the impact of COVID-19 on health care workers | USA | Letter to editor/  Opinion/ Discussion | HCWs | Describing effective measures to counteract the effects of the Covid-19 pandemic on the mental health of health workers | - Provide mobile health applications to screen the mental health of HCWs and refer them to centers - Hold multiple workshops to increase HCWs resilience - Assign a hot-line to communicate with psychologists |
| 10 | Supporting Clinicians during Covid-19 and Beyond — Learning from Past Failures and Envisioning New Strategies | Not mentioned | Letter to editor/  Opinion/ Discussion | HCWs | Investigating the psychological support strategies of health workers in the face of Covid-19 | - Promote peer support - Provide psychological services - Create a safe environment to hear HCWs fears and concerns - Eliminate the root cause of HCWs’ anxiety and stress, rather than relieving its symptoms - Actively get feedback and solve problems |
| 11 | Strategies for Resiliency of Medical Staff During COVD-19 | Not mentioned | Letter to editor/  Opinion/ Discussion | HCWs | Providing strategies to increase the resilience of HCWs in the face of Covid-19 | - Create an environment for effective communication between HCWs - Promote peer support - Create an hot-line to express psychological problems of HCWs and receive training such as stress management techniques |
| 12 | Supporting Hospital Staff During COVID-19: Early Interventions | Not mentioned | Letter to editor/  Opinion/ Discussion | HCWs | Explaining strategies to reduce burnout, increase resilience, improve mental health and relieve stress in HCWs | - Establish effective communication with HCWs to provide the required information - Periodically change HCWs from high-stress to low-stress departments and vice versa - Provide training about whatever staff may need and beyond clinical tips - Identify people who are more vulnerable and pay special attention to them - Create a sense of cohesion and unity among colleagues - Promote peer support |
| 13 | Well-Being During Coronavirus Disease 2019:  A PICU Practical Perspective | UK | Letter to editor/  Opinion/ Discussion | HCWs | Sharing executive changes made to the PICU to improve HCWs well-being | - Provide welfare facilities for HCWs - Creating a safe and separate space to relieve work-related stresses - Consider the space and time for HCWs to rest - Maintain and strengthen positive energy among HCWs - Provide space for HCWs to rest - Promote Peer support |
| 14 | Psychological Support System for  Hospital Workers During the Covid-  19 Outbreak: Rapid Design and  Implementation of the Covid-Psy  Hotline | France | Original | HCWs | Designing and implementation of psychological support system (Covid-Psy hotline) for hospital staff during Covid-19 | - Establish a hotline for psychological support of HCWs |
| 15 | Action Steps Toward a Culture of Moral Resilience in the Face of COVID-19 | USA | Letter to editor/  Opinion/ Discussion | Nurse | Providing peer support to improve moral resilience | - Make recommendations to improve the welfare and resilience of HCWs - Hold breathing sessions to relieve work pressure - Supply personal protective equipment |
| 16 | Resilience strategies to manage psychological distress amongst healthcare workers during the COVID-19  pandemic: a narrative review | Not mentioned | Review | HCWs | Summarizing strategies to increase the resilience of HCWs in conflict with Covid-19 | - Improve confidence in HCWs in that the organization will support them - Provide the necessary trainings - Provide facilities to solve the psychological problems of HCWs - Hold meetings aimed at training to increase HCWs' resilience |
| 17 | COVID-19 anxiety among front-line nurses: Predictive role of  organizational support, personal resilience and social support | Philippines | Original | Nurses | The effect of personal resilience, social support, and organizational support on reducing Covid-19 anxiety in order to strengthen frontline nurses | - Perform interventions to reduce HCWs’ anxiety by emphasizing the three factors of personal resilience, organizational support, and social support |
| 18 | The Bulle: Support and prevention of psychological decompensation of healthcare  workers during the trauma of the COVID-19 epidemic | France | Guideline/ report | HCWs | Development of a supportive program to prevent the psychological consequences of Covid-19 | - Consider a place for HCWs to talk and communicate - Develop peer support - Reorganize tasks to reduce the workload - Hold Schwartz rounds - Meet the welfare needs of HCWs - Establish honest communication between the management and HCWs |
| 19 | Predictors of Nurses’ Intentions to Work During the 2009 Influenza A (H1N1) Pandemic | USA | Original | Nurses | Evaluating predictors of nurses’ intentions for work during the Influenza A Pandemic | - Supply of personal protective equipment - Consider the health of nurses and their families |
| 20 | Staffing with Disease-Based Epidemiologic Indices  May Reduce Shortage of Intensive Care Unit Staff  During the COVID-19 Pandemic | Not mentioned | Original | HCWs | Providing solutions to reduce staff shortages by considering the epidemiology of a disease in staff scheduling | - Use staffing models that takes the epidemiology of a disease into account to reduce shortage of HCWs during pandemic - Provide appropriate equipment to prevent cross-contamination - Train in physical and psychological self-care - Monitor the health status of HCWs and quarantine infected individuals |
| 21 | COVID-19 and telemedicine: Immediate action required for maintaining  healthcare providers well-being | Not mentioned | Letter to editor/  Opinion/ Discussion | HCWs | Review of telemedicine and its applications | - Usetelemedicine to reduce the workload and the possibility of infection of the HCWs |
| 22 | Using a m-Health tutorial application to  change knowledge and attitude of frontline  health workers to Ebola virus disease in  Nigeria: a before-and-after study | Nigeria | Original | HCWs | Evaluating the effect of using a mobile training application to improve the knowledge and attitude of health workers to Ebola virus | - Use training application to educate HCWs about the disease and ways to prevent its transmission and self-care |
| 23 | Preserving mental health and resilience in frontline healthcare workers during COVID-19 | Not mentioned | Letter to editor/  Opinion/ Discussion | Physicians | Providing solutions to support physicians during Covid-19 | - Provide adequate personal protective equipment - Meet welfare needs - Family and community support of doctors - Psychological support for physicians due to their personality differences - Prepare retired staff, students, the army, etc. to help the medical staff |
| 24 | Quality improvement report: setting up  a staff well-being  hub through  continuous engagement | United Kingdom | Original | HCWs | Reviewing the experience of setting up a staff well-being hub with a serene environment to enable psychological resilience during the Covid-19 pandemic | - Establish a center to support HCWs and to reduce their work stress - Enable interaction and peer support between HCWs |
| 25 | Psychological distress, coping behaviors, and preferences for support among New York healthcare workers during the COVID-19 pandemic | USA | Original | HCWs | Assessing mental health among HCWs and identifying distress among them, and providing selected solutions to support them | - Engage HCWs in sports, yoga, meditation, faith-based religion, and spirituality - Talk therapy and virtual provider support groups |
| 26 | e-ICU's/Tele ICU's, its Role, Advantages Over Manual ICU's and Shortcomings in the Current Perspective of Covid-19 Pandemic: A critical Review | Not mentioned | Letter to editor/  Opinion/ Discussion | Health Care Workers | The Role and advantage of Tele-ICUs in reducing the chance of infection of health care workers | - Use Tele-ICU to reduce the exposure of health care workers |
| 27 | Coping With Trauma, Celebrating Life: Reinventing Patient And Staff Support During The COVID-19 Pandemic | USA | Letter to editor/  Opinion/ Discussion | Health Care Workers | Providing supportive solutions to meet the challenges of health care workers and patients | - Organize a psychological team to help HCWs - Create a restroom - Temporary lodge suspicious staffs in hotels - Provide facilities such as childcare services, transportation, and meals. - Hold a recovery party for patients. - Create a mourning room to keep the memory of deceased HCWs |
| 28 | Attending to the Emotional Well-Being of the Health Care Workforce in a New York City Health System During the COVID-19 Pandemic | USA | Letter to editor/  Opinion/ Discussion | Health Care Workers | Strategies for promoting and maintaining the welfare of health workers | - Meet basic needs such as food, housing, and transportation Providing facilities such as childcare services - Strengthen communication networks with HCWs for psychological support |
| 29 | Challenges and solutions for addressing critical shortage of supply chain for personal and protective equipment (PPE) arising from Coronavirus disease (covid-19) pandemic-Case study from the Republic of Ireland | Republic of Ireland | Original | Health Care Workers | Challenges and solutions to the lack of personal protective equipment for HCWs in the Covid-19 pandemic | - Reprocess PPE - Use various communication channels to provide PPE - Bespoke production of hospital equipment using modern and low-cost methods |
| 30 | Do Shared Barriers When Reporting to Work During an Influenza Pandemic Influence Hospital Workers’ Willingness to Work? A Multilevel Framework | USA | Original | Health Care Workers | Relationship between the characteristics of health system’s HCWs and interventions and barriers affecting their desire | - Provide financial benefits - Consider specific time off - Provide drug and PPE for HCWs and their families |
| 31 | Leveraging Cloud Based Virtual Care as a Tool Kit for mitigating Risk of Exposure during a Pandemic | Not mentioned | Letter to editor/  Opinion/ Discussion | Health Care Workers | Virtual care through telemedicine as a tool to reduce the chances of infection | - Initial screening of suspicious cases through video calls and virtual service kiosks in local centers |
| 32 | Maximizing the Resilience of Healthcare Workers in Multi-hazard Events: Lessons from the 2014–2015 Ebola Response in Africa | Africa | Letter to editor/  Opinion/ Discussion | Health Care Workers | Describing the APD model and its integration with the PsySTART-R model and its operational implementation | - Use APD model and PsySTART-R checklist to monitor stress and develop personal resilience programs for HCWs |
| 33 | COVID-19 in Africa: care and protection for frontline healthcare workers | Africa | Review | Health Care Workers | Providing solutions to support HCWs to reduce the risk of infection and mental health of HCWs | - Supply PPE - Train the staff in the use of PPE - Provide vaccines and laboratory tests for staff |
| 34 | The dental team as part of the medical workforce during national and global crises | Not  mentioned | Letter to editor/  Opinion/ Discussion | Dentists | Evidence of dentists' empirical knowledge, communication and clinical skills to join the treatment team during the Covid-19 pandemic | - Use dentists as a member of the treatment team to reduce the workload |
| 35 | Computer-assisted resilience training to prepare healthcare workers for pandemic influenza: a randomized trial of the optimal dose of training | Canada | Original | Health Care Workers | The effectiveness of online training to increase the resilience of HCWs in pandemic conditions | - Implement online training courses to increase HCWs resilience |
| 36 | The Witness to Witness Program: Helping the Helpers in the Context of the COVID-19 Pandemic | USA | Original | Health Care Workers | Describing the W2W model and its supportive role in reducing HCWs’ anxiety in stressful situations such as Covid-19 | - Hold webinars, telephone counseling sessions - Develop an individual program to reduce the anxiety |
| 37 | COVID-19 Peer Support and Crisis Communication Strategies to Promote Institutional Resilience | Not mentioned | Letter to editor/  Opinion/ Discussion | Health Care Workers | Strategies to boost resilience and HCWs’ well-being during Covid-19 pandemic | - Establish a communication network between managers and HCWs - Develop peer Support |
| 38 | The COVID-19 Pandemic and the Health Care Providers; What Does It Mean Psychologically? | Not mentioned | Review | Health Care Workers | Symptoms of HCWs’ psychological stress and its management strategies | - Develop realistic plan to reduce the workload - Establish honest communication with HCWs - Develop peer support - Organize a psychological team to help HCWs - Establish a communication network for sharing successful experiences and the most up-to-date information - Meet the training needs of HCWs - Use APD and Folkman & Greer models to increase HCWs’ resilience |
| 39 | A psychological health support scheme for medical teams in COVID-19 outbreak and its effectiveness | China | Original | Health Care Workers | Design and evaluation of psychological support model for treatment staff during the Covid-19 pandemic | - Initial assessment of the psychological status of HCWs - Send motivational messages to HCWs - Hold counseling sessions - Create special support teams to advise HCWs |
| 40 | Applying the Lessons of SARS to Pandemic Influenza An Evidence-based Approach to Mitigating the Stress Experienced by Healthcare Workers | Not mentioned | Letter to editor/  Opinion/ Discussion | Health Care Workers | Providing an evidence-based approach to build resilience and reduce the stress of HCWs | - Strengthen the personal resilience of HCWs through psychological screening and efforts to improve their mental state - Provide psychological first aid training - Consider alternative resources and planning - Delegate authority to make decentralized decisions - Develop effective leadership of managers in the implementation of programs |
| 41 | Chapter6. Protection of patients and staff during a pandemic | Not mentioned | Letter to editor/  Opinion/ Discussion | Health Care Workers | Preparation of suggestions and performance standards in the intensive care unit to prepare hospitals to face crises and pandemics with emphasis on protecting patients and staff | - Consider specific means of transportation for staff - Provide the PPE for HCWs and their families - Rearrangement of HCWs’ shifts to reduce their workload - Reduce patient admissions in order to control work pressure on staff - Use clinical workers in other departments to reduce the workload - Allocate drugs and vaccines to both the HCWs and their families - Provide leave for HCWs to mourn their recently deceased relatives in the epidemic - Support staff in the event of work-related violence between health workers and patients' visitors |
| 42 | Burnout and Posttraumatic Stress Disorder in the Coronavirus Disease 2019 (COVID-19) Pandemic: Intersection, Impact, and Interventions | Not mentioned | Original | Medical doctors | Providing a concept map to show the relationship between burnout, acute and post-traumatic stress disorder, and provide system-oriented interventions to reduce physicians’ stress. | - Teach meditation to HCWs - Use modern treatment techniques to deal with psychological consequences - Plan in order to reduce clinical and Face-to-face visit - Create a space to promote the team spirit of HCWs - Improve staff training in order to increase their skills and mastery of specialized matters - Encourage HCW participation in the process of planning and organizational decisions - Providetelemedicine infrastructure for radiologists to work from home - Educate counselors and psychologists through panel discussions with specialists about treating common staff problems such as burnout, PTSD - Facilitate HCWs' access to information related to their mental health - Make efforts to reduce social stigmas - Use novel methods (narrative medicine) in order to play an active role of physicians in their treatment process - Strengthen the spirit of sacrifice and altruism of HCWs - Explicit communication by the management with HCWs to increase the feeling of security, teamwork, stability, and endurance of HCWs |
| 43 | Containment of COVID-19 cases among healthcare workers: The role of surveillance, early detection, and outbreak management | Singapore | Original | Health Care Workers | Design and implementation of a comprehensive, integrated strategy for early detection of Covid-19 among the hospital staff | - Provide infection control and early detection of HCWs using a 3-step process based on: - Self-declaration of HCWs who presented symptoms - Quick tracking of people in contact with an infected person - Performing testing and starting treatment and quarantine for patients |
| 44 | Igniting Change Supporting the Well-Being of Academicians Who Practice and Teach Critical Care | Not mentioned | Review | Nurses | Introducing appliable model to improve the conditions medical attendees working in the ICU | - Pay attention to the physical health of HCWs by paying attention to their sleep, exercise, and nutrition - Strengthen HCWs' social relationships with each other - Initiate joint activities such as a book club, a joint lunch or dinner for staff outside the workplace - Increase HCWs' self-awareness about the symptoms of stress or depression and follow it up as needed |
| 45 | Keep Calm and Log On: Telemedicine for COVID-19 Pandemic Response | USA | Letter to editor/  Opinion/ Discussion | Medical doctors | A summary of the practical applications of telemedicine | - UseVirtual Practicing to screen and treat patients and reduce physicians' exposure - Compensate for staff shortages |
| 46 | Operative team checklist for aerosol generating procedures to minimize exposure of healthcare workers to SARS-CoV-2 | Not mentioned | Original | Health Care Workers | A structured approach for surgical teams to control producted aerosols and minimize treatment team exposure to them | - Provide a checklist to minimize HCWs’ exposure during the Covid-19 pandemic |
| 47 | Mental health support to staff in a major hospital in Milan (Italy) during the COVID-19 pandemic: a framework of actions | Italy | Letter to editor/  Opinion/ Discussion | Health Care Workers | Presenting a set of measures to support the mental health of HCWs during the Covid-19 pandemic in a hospital in Milan, Italy | - Hold online training courses for HCWs - Teach training tips to HCWs with the help of the intranet or internet and personal phones of personnel - Allocate a place for HCWs to rest in a place close to the workplace with easy access to water and food and etc. - Provide information about how to reduce stress and increase mental flexibility - Send supportive and motivational messages from prominent patients, managers, and social and national activists with supportive themes (# You-are-not-alone) - Provide easy access to the hot-line for psychiatric, counseling, and medication support - Encourage commitment of managers to provide medical services for HCWs - Do online group meditation exercises - Contact and communicate with quarantined staff who are ill - Put QR codes in the departments for easy and immediate access of HCWs to information and receiving various supports - Encourage regular visits of managers to check and compensate for human resources and PPE deficiencies - Hold group therapy sessions for staff - Exchange experiences globally |
| 48 | Supporting nurses’ mental health  during the pandemic | Not mentioned | Letter to editor/  Opinion/ Discussion | Health Care Workers | The Effects of Organizational policies in the field of PPE and visits of patients' visitors on nurses' mental health and practical suggestions for maintaining nurses' mental health | - Provide PPE for patients' visitors - Provide smartphones or tablets for each patient in order to maintain the patient's remote connection with their families - Transfer palliative care specialists to inpatient wards of Covid-19 patients - Teach the principles of palliative care medicine to HCWs at the forefront of health care systems - Allocate non-nurse staff as intermediaries between patients and their families to reduce the workload of nurses - Teach HCWs the principles of mental health - Provide financial support for nurses - Establish mechanisms to assess and evaluate the mental health of HCWs - Assessment of HCWs' mental condition through face-to-face interviews by managers - Create a positive atmosphere in order to increase the relationship between HCWs with each other - Consider a hotline for providing information on staff needs, including financial, equipment, and emotional needs - Consider specialized teams to treat injured people |
| 49 | Screening of healthcare workers for SARS-CoV-2 highlights the role of asymptomatic carriage in COVID-19 transmission | United Kingdom | Original | Health Care Workers | Investigating the need to screen asymptomatic HCWs | - Screening the asymptomatic HCWs in order to prevent patients and other HCWs |
| 50 | Enhancing Psychological Sustainment & Promoting Resilience in Healthcare Workers During COVID-19 & Beyond: Adapting Crisis Interventions from High-Risk Occupations | Not Mentioned | Review | Health Care Workers | Discusses current and emerging literature on the unique impacts of COVID-19 on HCWs and provides actionable, evidence-informed recommendations for individuals, teams, and leaders to enhance sustainment of HCWs that is critical to the preservation of national and global health security. | - Helping individuals to increasing Self-Care & Self-Monitoring like attention to quality of their Sleep, nutrition, hydration, and exercises beside their vital signs - Limit exposure to disaster related and other negative media - Practical Supports, like Food, parking, lodging, and child-care are essential for HCWs - Procedures to reduce infection risk of HCWs’ safety - Providing adequate supplies of effective equipment to protect HCWs - Efforts to enhance connections among personnel due to truthful messages create realistic atmosphere for sharing additional information and enhancing trust in organizations - Giving information about when and where to get help through timely, realistic, and updated training - Educating about growth mindset - Leaders should use effective communication by knowing and practicing effective strategies - Leaders should Helping the work community by make meaning of the event, and then helping personnel to look hopefully to the future |
| 51 | An Executive Strategy to Support Long-Term Clinician Engagement Amid the COVID-19 Pandemic | Not Mentioned | Letter to editor/ Opinion/ Discussion | Health Care Workers | Outlining the 5 actions that executives should take to bolster staff engagement and their resilience in a long-term period according to COVID-19 pandemic. | - Ensuring that staff are safe and feel safe when working (PPE + greater level of transparency with frontline staff about who is making decisions on safety standards, what clinical evidence is guiding each decision, and when and how staff can expect updates) - Rapid decision making over collecting input from a wide variety of frontline clinicians (recruitment of new employee) - Transitioning the leaders from sprint mode to marathon mode (Executives must help managers deprioritize non- essential projects to reduce workload and encourage managers to consciously take time to invest in their own ongoing well-being) |
| 52 | Enhancing Nurse Manager Resilience in a Pandemic | USA | Original | Nurses | increasing self-reported resilience in the group Nurses from 3 regional hospitals over a 6-month period using an automated web-based tool (WISER as standing for Web-based Implementation of the Science of Enhancing Resilience) | - The WISER tool shows promise as a means of enhancing resilience. In this method nurse managers received an automated daily text message with a hyperlink to the website where different resilience enhancing tools were introduced. - Nurse leaders should share creative, successful strategies to build community and support for the nurses, especially during the very stressful times |
| 53 | Rapid adoption of resilience strategies during the COVID-19 pandemic | Georgia | Letter to editor/ Opinion/ Discussion | Health Care Workers | How the Medical Association of Georgia is adapting and using “Diffusion of Innovation” techniques to formulate guidelines and help Georgia’s medical facilities implement evidence-based practices during the pandemic | - Using Diffusion of innovation (DOI) model about creation of an innovation to widespread practice is effective. (It’s included five stages of knowledge, persuasion, decision, implementation, and confirmation) |
| 54 | Burnout Syndrome Prevention in Nursing at Pandemic (Covid-19): A Literature Review | Not Mentioned | Review | Nurses | To identify prevention strategies to decrease burnout syndrome in nursing personnel in times of pandemic COVID-19 on existing evidences | - Organizational Strategies, which are subdivided into 9 aspects of mental and physical health, work environment, teamwork, work shift, support groups, online assistance, training, structure/operation, and health policies. - Personal Strategies which focused on communication, self-care, and stress management technique |
| 55 | Resilience strategies to manage psychological distress among healthcare workers during the COVID-19 pandemic | Australia | Review | Health Care Workers | Management strategies to increase resilience in healthcare workers during the COVID-19 pandemic | - Holding daily exercise programs - Help to Sleep hygiene of the HCWs - Step toward to performing social support of HCWs - Improve Meaning of work by small group discussions & reﬂective counselling sessions - Mindfulness practice - Stress management programs - Organizational justice - Competency Training - Computer based resilience training - Education sessions (resilience focused) - Anticipate, plan and deter responder risk and resilience model - Psychological ﬁrst aid - Staff feedback sessions |
| 56 | Interventions to support the resilience and mental health of frontline health and social care professionals during and after a disease outbreak, epidemic or pandemic - a mixed methods systematic review | Not Mentioned | Review | Health Care Workers | How successfully any interventions improved frontline health professionals’ resilience or mental well-being; And, identify barriers and facilitators that may affect the implementation of interventions to support resilience and mental health of front-line health and social care professionals in the event of an outbreak, epidemic or epidemic | - Workplace structure and routine interventions, for example, regular breaks, shorter working hours, regular team meetings, mentorship, relaxation or recreation areas in workplaces - Provision of information, guidance, or training, for example, on dealing with diﬀicult situation - Promoting or supporting healthy lifestyle and self-care like eating, sleeping, exercising, following a routine, avoiding excess social media, staying in touch with family and friends, engaging in enjoyable activities - Relaxation techniques, for example, progressive muscle relaxation, meditation - Therapist-delivered psychological interventions, delivered individually or in groups, and face-to-face or by text or video call, including professional psychological or counselling support, CBT and psychotherapy - Guided self-help strategies, such as online CBT, online/web well-being and sleep apps, and mindfulness programs. For inclusion, guided interventions had to describe the type of support oﬀered (e.g. telephone, online, video) - Non-guided self-help strategies, such as online/computer, audio or book-based self-guided interventions (these can also include self-guided CBT, mindfulness, mediation, and exercises such as writing down worries) - Workplace-based psychological support strategies, such as peer support networks, employee wellness programs, and psychological first aid - Medication for depression, anxiety, sleep disorders, or a combination of any or all of these |
| 57 | Building resilience for healthcare professionals working in an Italian red zone during the COVID-19 outbreak | Italy | Original | Health Care Workers | Establishing the pilot implementation of the R2 model as a resilience curriculum for healthcare leaders dealing with the COVID-19 pandemic, with the goal of improving psychological health and enhancing individual and work team resilience | The “R2 for Leaders” model is consisted of rugged qualities and external resources. It is an evidence‐informed resilience promoting curriculum for organizations that built on promising practices that have been shown to enhance wellbeing among individuals experiencing stress and adversity. Rugged qualities focused on:   - Gratitude - Self‐confidence - Optimism - Problem‐solving - Mindfulness - Sleep - Nutrition - Physical daily activity   External resources focused on:   - General justice structure of the organization - Helping individuals to recognize the importance of duties and their responsibilities - Supportive relationships - A powerful identity of individuals & experiences of authority - Fair treatment - Meeting of basic needs |
| 58 | Promoting resilience in healthcare workers during the COVID-19 pandemic with a brief online intervention | US | Original | Health Care Workers | This study aimed to evaluating the quantitively results of feasibility and acceptability of a brief online course focused on introducing evidence-based skills that could increase resilience and decreases emotional distress in HCWs during the pandemic | The online course, called Resilience Training (RT) model consists of three short videos that focused on mindfulness, mentalization, and self-compassion which are evidence-based skills or capacities that shown to maintain or increase aspects of emotional resilience. The RT includes:   - Didactic information - Experiential exercises & - Testimonials from HCWs |
| 59 | Practical strategies and the need for psychological support: recommendations from nurses working in hospitals during the COVID-19 pandemic | US & Canada | Letter to editor/  Opinion/ Discussion | Nurses | The goal of this study was to capture the recommendations of nurses providing frontline care during the pandemic due to mitigate stressful working conditions | - A leadership style that embodied visibility, availability and careful planning - A clear, consistent and transparent communication due to information overload - Resilient healthcare supply chain (PPE & etc.) - Clear communication of policies related to sick leave, pay equity and workload - Equity should be considered, particularly with regard to redeployment - Psychological support offered by trusted providers, managers and peers |
